# Effect of desmopressin administration on intraoperative blood loss and quality of the surgical field during functional endoscopic sinus surgery: a randomized, clinical trial

**DOI:** 10.1186/s12871-015-0034-8

**Published:** 2015-04-17

**Authors:** Hua Shao, Li-Ting Kuang, Wei-Jian Hou, Tao Zhang

**Affiliations:** 1 Department of Anesthesiology, The First Affiliated Hospital, Sun Yat-sen University, Guangzhou, 510080 China; 2 Department of Anesthesiology, The First Affiliated Hospital of Zhengzhou University, Zhengzhou, 450000 China; 3 Otorhinolaryngology Hospital, The First Affiliated Hospital of Sun Yat-sen University, Guangzhou, 510080 China

**Keywords:** Desmopressin, Blood loss, Surgical field, Endoscopic sinus surgery

## Abstract

**Background:**

Bleeding during functional endoscopic sinus surgery is a challenge for the quality of the surgical field for surgeons. This study aimed to evaluate the effect of desmopressin premedication on blood loss and the quality of the surgical field in endoscopic sinus surgery.

**Methods:**

A total of 90 American Society of Anesthesiologists physical status I–II patients underwent endoscopic sinus surgery for chronic sinusitis. They were randomly allocated to receive either desmopressin 0.3 μg/kg or saline before the operation. Management of anesthesia was achieved with propofol and remifentanil infusions, with moderate, controlled hypotension. Blood loss and quality of the surgical field were assessed after surgery. Effects of desmopressin on anesthetic requirements and hemodynamic variables were analyzed.

**Results:**

Blood loss was significantly less in the desmopressin group (mean ± SD, 42 ± 8.7 ml) than in the control group (70 ± 9.2 ml, *P* < 0.001). Surgeons were more satisfied with the surgical field in the desmopressin group than in the control group (median score, 4 [3–5] vs. 7 [6–9], *P* < 0.001). Requirements for remifentanil and esmolol were lower in the desmopressin group than in the control group.

**Conclusions:**

Premedication with desmopressin 0.3 μg/kg can effectively reduce bleeding during endoscopic sinus surgery.

## Background

Functional endoscopic sinus surgery (FESS) is a common technique for surgical management of chronic rhinosinusitis [1]. Although major blood loss during FESS is rare, even a small amount of blood may disturb the surgical view. This disturbance prolongs the operation time, increases the likelihood of complications, and possibly results in incomplete surgery [2]. Several techniques have been suggested to improve the surgical field in endoscopic sinus surgery, such as bipolar diathermy, topical vasoconstrictors, local injection of epinephrine, and induced hypotension [3,4]. However, none of these techniques have consistently provided an ideal surgical field for the surgeon. Bleeding in FESS remains a challenge for surgeons and anesthesiologists.

Desmopressin (1-deamino-8-D-arginine vasopressin) has been used as a treatment for mild to moderate hemophilia and von Willebrand’s disease. Desmopressin has also been used to reduce the prolonged bleeding time that often accompanies uremia, hepatic cirrhosis, and other causes of platelet dysfunction. Hemostatic effects of desmopressin include increasing plasma concentrations of coagulation factor VII, von Willebrand factor, and tissue plasminogen activator; improving platelet adhesiveness; and reducing bleeding time [5]. Several studies have been performed on patients undergoing various types of surgeries. A study in patients undergoing lumbar fusion showed that desmopressin was effective in reducing intraoperative blood loss when the anticipated bleeding exceeded 1 liter [6]. Another study in patients undergoing orthognathic surgery showed a significant reduction in intraoperative blood loss [7]. However, the effect of desmopressin administration on intraoperative blood loss in endoscopic sinus surgery is unknown.

In addition to the effects desmopressin exerts on hemostasis, it can also induce hypotension [8]. There is general consensus that when FESS is performed under general anesthesia, maintaining moderate, controlled hypotension (mean arterial blood pressure [MAP], 60–70 mm Hg) is important for improving surgical visibility. This improvement results in faster surgery and a reduced risk of complications, such as massive hemorrhage, skull base defects, and blindness [9,10]. Permissive hypotension and desmopressin have additive effects in reducing blood loss for uncontrolled hemorrhage [11]. Although the mechanism of hypotension is unclear, desmopressin may have combined beneficial effects on hemostasis and control of hypotension, and thus on blood loss in endoscopic sinus surgery patients. This study aimed to determine the effect of administration of desmopressin on intraoperative blood loss and the surgical field in patients undergoing endoscopic sinus surgery.

## Methods

We prospectively studied 90 American Society of Anesthesiologists physical status I and II patients with chronic rhinosinusitis presenting for bilateral FESS. The trial was registered after the beginning of the study (NCT 02125188). The study was approved by the Medical Ethics Committee of the First Affiliated Hospital of Sun Yat-sen University, Guangzhou, China. Written informed consent was obtained from the patients.

All patients were 18–65 years old, and were first-time candidates for two-side FESS. Specific exclusion criteria were as follows: patients with a history of bleeding disorders or taking medications that may affect surgical hemostasis; secondary surgery; poorly controlled hypertension or cerebrovascular disease; a history of significant coronary artery disease or arrhythmias; a compromised renal or hepatic function; and pregnancy. Patients were randomly assigned to one of two study groups: patients in the desmopressin group received 0.3 μg/kg of desmopressin preoperatively and those in the control group received normal saline instead. Assignment to the groups was performed by computer-generated random numbers.

All patients had a normal preoperative platelet count and coagulation test results. Anesthetic management of all patients was standardized and conducted by a single attending anesthesiologist. All of the surgeries were performed by a single, experienced surgeon. All of the surgeons were blinded to the patient study groups.

Patients’ potential surgical predictors of intraoperative blood loss were recorded before surgery. These predictors included the presence of sinonasal polypoid disease and endoscopically documented active infection, computed tomography-graded severity of sinus disease based on the Lund–MacKay scoring system [12], a history of previous endoscopic sinus surgery, and a history of taking oral steroids within 2 weeks before surgery.

General anesthesia was induced with intravenous (IV) fentanyl 3 μg/kg and propofol 2 mg/kg. Tracheal intubation was facilitated with IV cisatracurium 0.2 mg/kg. Intermittent positive pressure ventilation was followed to maintain a normal end-tidal carbon dioxide partial pressure. Shortly after induction, patients in the desmopressin group received 0.3 μg/kg of desmopressin that was diluted in 100 ml of normal saline. Patients in the control group received 100 ml of normal saline IV over 20 min.

Anesthesia was maintained with bispectral index-adjusted continuous IV infusions of propofol (50–140 μg /kg/min) and remifentanil (0.15–0.3 μg/kg/ min), to maintain MAP within 60–70 mmHg, as measured on the patient’s arm every 5 min. Acute increases in MAP were treated with additional boluses of IV remifentanil (0.5–1 μg/kg) and escalating doses of IV esmolol (10–20 mg), followed by incremental doses of IV nicardipine (0.2–0.4 mg). Heart rate (HR) was recorded every 5 min. Propofol and remifentanil infusions were discontinued 10–15 min before the end of the surgery. The duration of surgery was recorded in all of the patients.

Local control of bleeding in the surgical field was facilitated by submucosal single time injection of epinephrine (1:100,000, 3 ml) by the surgeon. Crystalloid solution was administered to replace overnight fluid deficits, provide maintenance fluid requirements, and replace blood loss on a 3:1 basis.

Final intraoperative blood loss was confirmed at the end of each procedure by accounting for loss of blood and irrigation fluid into the suction canister, the patient’s stomach (measured volume of gastric contents), and nasopharyngeal packing (measured weight of packing on the electronic scale). The overall quality of the surgical field was assessed at the end of each procedure by the surgeon according to a scoring scale adapted from Boezaart et al. [9] (Table 1). Possible side effects of desmopressin, such as myocardial infarction, cerebral infarction, and other thromboembolic complications, were investigated by the surgeon.

**Table 1:** **Quality of the intraoperative surgical field during functional endoscopic sinus surgery**

0–1	No bleeding; excellent to outstanding surgical conditions
2–3	Slight bleeding; surgery fairly easy. No stops for hemostasis and/or suctioning are required
4–5	Slight bleeding; surgery mildly difficult. One stop for hemostasis and/or suctioning is required
6–7	Moderate bleeding; surgery moderately difficult. Occasional stops for hemostasis
8–9	Moderate to severe bleeding; surgery very difficult. Multiple stops for hemostasis
10	Surgery terminated due to severe bleeding in the surgical field

We considered a 30% reduction in blood loss to be clinically significant. To demonstrate this with a power of 80% at the 0.05 level of significance, we calculated that 45 patients would be required in each group. Results are expressed as mean, standard deviation, median, and range. Parametric data were compared using the Student’s *t*-test and non-parametric data were analyzed with the chi-squared test and Mann–Whitney *U* test. All statistical calculations were performed with SPSS 16.0. *P* < 0.05 was considered significant.

## Results

**Figure 1:**
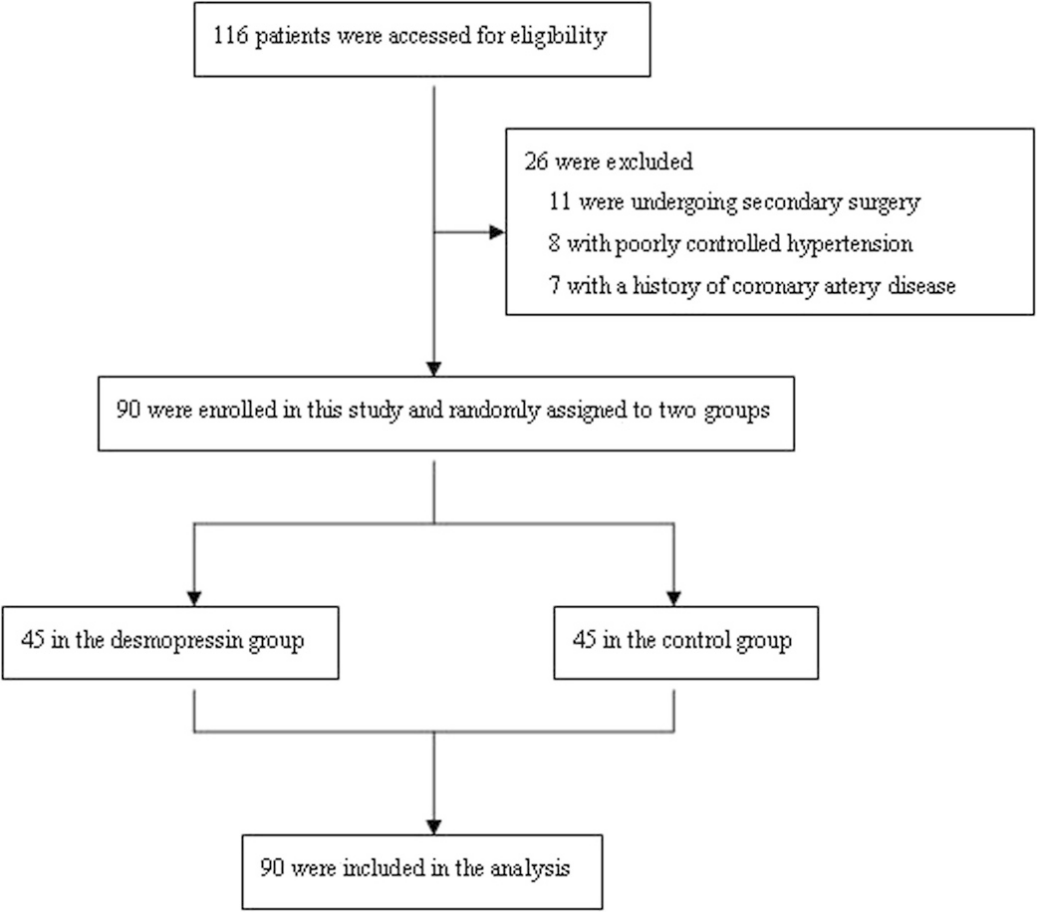
Flow chart. The chart shows that 116 patients were initially screened for the study and finally 90 patients were included in the data analysis.

**Figure 2:**
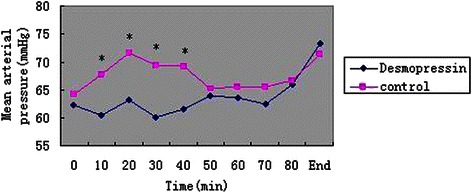
Mean arterial blood pressure values in each of the groups over the course of surgery. The symbols show mean values. Data are presented at 10-min intervals, up to 80 min. The “0” and “End” values refer to the start and end of surgery, respectively. *P < 0.05 compared with the control group.

**Figure 3:**
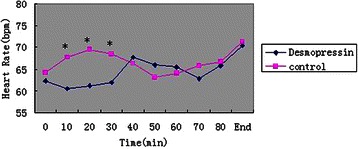
Heart rate in each of the groups over the course of surgery. The symbols show mean values. Data are presented at 10-min intervals, up to 100 min. The “0” and “End” values refer to the start and end of surgery, respectively. *P < 0.05 compared with the control group.

A total of 90 patients were recruited, and all of the patients completed the study (Figure 1). All of the groups were comparable with respect to demographic characteristics and all surgical covariates (Table 2). There was a significant effect of desmopressin on blood loss and quality of the surgical field (Table 2). Although there was a significant effect of treatment on MAP (Figure 2) and HR (Figure 3) at several time points, mean MAP and HR differences were small and transient.

**Table 2:** **Patient’s demographic characteristics and characteristics of functional endoscopic sinus surgery**

	Desmopressin (n = 45)	Control (n = 45)	*P*
Age (yr)	45 (14.9)	40.7 (17.4)	0.2
Weight (kg)	62.7 (16.7)	59.0 (16.1)	0.3
Gender (M/F)	26/19	24/21	0.7
Surgical blood loss (mL)	42 (8.7)	70 (9.2)	<0.001
Quality of surgical field (0–10)	4 (3–5)	7 (6-9)	<0.001
Duration of surgery (min)	76 (20.7)	82 (19.6)	0.2
Polypoid disease (Y/N)	34/11	32/13	0.6
CT score (0–24)	15 (11–18)	13 (10–18)	0.6
Active infection (Y/N)	9/36	8/37	0.8
Preoperative steroids (Y/N)	16/29	11/34	0.3
Revision surgery (Y/N)	12/33	9/36	0.5

Data expressed as mean (SD) or median (interquartile range) where appropriate.

While the total dose of IV propofol and nicardipine was not different between the study groups, patients in the desmopressin group showed lower requirements for IV remifentanil and esmolol than those in the control group (Table 3). There were no postoperative thromboembolic complications in any group.

**Table 3:** **Perioperative anesthetic and antihypertensive drug requirements during functional endoscopic sinus surgery**

	Desmopressin (n = 45)	Control (n = 45)	*P*
Propofol (mg)	830 (195)	780 (177)	0.2
Remifentanil (μg)	1350 (235)	1785 (260)	<0.001
Esmolol (mg)	15 (4.5)	40 (7.5)	<0.001
Nicardipine (mg)	0.25 (0.06)	0.26 (0.07)	0.5

Data expressed as mean (SD).

The duration of surgery was shorter in the desmopressin group than in the control group, but this difference was not significant.

## Discussion

Our study showed that preoperative desmopressin administration reduced blood loss and improved the quality of the surgical field during FESS. These findings indicated the potential clinical benefit of desmopressin under our study conditions.

Several techniques have been suggested to reduce bleeding in endoscopic sinus surgery. Wormald et al. showed that injection of the pterygopalatine fossa resulted in an improved surgical field during FESS [4]. Boezaart et al. compared sodium nitroprusside- and esmolol-induced, controlled hypotension for FESS, and found that the esmolol group showed superior surgical conditions compared with the nitroprusside group [9]. Another study showed that preoperative administration of oral clonidine reduced intraoperative blood loss and improved quality of the surgical field [13]. The present study showed the efficacy of premedication with desmopressin in reducing blood loss in FESS. This finding may be attributed to the hemostatic effects of desmopressin, which include an increase in plasma coagulation factor VII, von Willebrand factor, and tissue plasminogen activator, and improvement of adhesiveness of platelets [5].

Proper anesthetic management can indirectly decrease blood loss during FESS by improving the operating conditions, which could result in a more rapid surgery [10]. Maintaining moderate, controlled hypotension is important for improving surgical visibility during FESS. Under the conditions of moderate, controlled hypotension, the quality of the surgical field is affected by achieving a reduction in HR, to promote superior hemodynamic stability and minimize direct peripheral vasodilation [14,15]. These strategies were successfully used in our study. There were no cases of considerable hypotension (decrease >30% from baseline MAP) induced by desmopressin in either of the study groups.

In our study, significantly smaller doses of IV remifentanil and esmolol were required in the desmopressin group than in the control group to maintain intraoperative MAP within the desired range (Table 3). This finding likely reflects the hypotensive effect of desmopressin [8] and negative inotropic and direct vasodilating effects of esmolol and remifentanil [9,16].

One limitation of this study was that we could not address the severity of sinus disease in our patients. However, all of the patients in this study were first-time candidates for two-sided FESS because of chronic sinusitis. In this study, we used a subjective scale to evaluate the quality of the surgical field, as well as satisfaction of the surgical team. The efficacy and reliability of this measurement have been verified [17].

Because all pre-coagulative drugs (including desmopressin) might have pro-thrombitic effects, the use of desmopressin should be under a risk-benefit calculation in patients with risk of hypercoagulability. Therefore, we excluded patients with poorly controlled hypertension or cerebrovascular disease, those with a history of significant coronary artery disease or arrhythmias, and those who were pregnant. And we didn’t find any postoperative thromboembolic complications among the patients.

## Conclusion

This study shows an independent effect of desmopressin administration on blood loss and quality of the surgical field during endoscopic sinus surgery that is performed under propofol and remifentanil anesthesia, and under moderate, controlled hypotension.
